# The analysis of the function, diversity, and evolution of the *Bacillus* phage genome

**DOI:** 10.1186/s12866-023-02907-9

**Published:** 2023-06-19

**Authors:** Yating Zhang, Jianjia Miao, Ning Zhang, Xiaoyu Wang, Zijing Li, Owusu Ansah Richard, Bingxue Li

**Affiliations:** 1 College of Bioscience and Biotechnology, Shenyang Agricultural University, Shenyang, 110866 China; 2 College of Land and Environment, Shenyang Agricultural University, Shenyang, 110866 China; 3 Food Science College, Shenyang Agricultural University, Shenyang, 110866 China

**Keywords:** *Bacillus*, Phages, Genome, Functions, Diversity, Evolution

## Abstract

**Background:**

Phages play a pivotal role in the evolution of microbial populations. The interactions between phages and their hosts are complex and may vary in response to host physiology and environmental conditions. Here, we have selected the genomes of some representative *Bacillus* prophages and lysosomes from the NCBI database for evolutionary analysis. We explored their evolutionary relationships and analyzed the protein information encoded by hundreds of *Bacillus* phages.

**Results:**

We obtained the following conclusions: First, *Bacillus* phages carried some known functional gene fragments and a large number of unknown functional gene fragments, which might have an important impact on *Bacillus* populations, such as the formation of spores and biofilms and the transmission of virulence factors. Secondly, the *Bacillus* phage genome showed diversity, with a clear genome boundary between *Bacillus* prophages and *Bacillus* lytic phages. Furthermore, genetic mutations, sequence losses, duplications, and host-switching have occurred during the evolution of the *Bacillus* phage, resulting in low genome similarity between the *Bacillus* phages. Finally, the lysis module played an important influence on the process of *Bacillus* phage cross-species infestation.

**Conclusions:**

This study systematically described their protein function, diversity, and genome evolution, and the results of this study provide a basis for evolutionary diversity, horizontal gene transfer and co-evolution with the host in *Bacillus* phages.

**Supplementary Information:**

The online version contains supplementary material available at 10.1186/s12866-023-02907-9.

## Background

The genus *Bacillus* is a kind of bacillary bacteria that exists widely in nature [1], and the dormant spores produced by *Bacillus* can survive in harsh circumstances (e.g., high temperature, desiccation, UV and γ-radiation) or even extraterrestrial conditions [2, 3]. Many *Bacillus* species produce bioactive molecules, including lipopeptides [4], polyketide compounds [5], bacteriocins [6], and siderophores [7] which are beneficial for plant health. Because of the production of these bioactive molecules, many *Bacillus* species are known to promote root growth, suppress pathogens, kill pests, and have cytotoxic effects on liver and colon cancer cells [8–10]. In addition, some *Bacillus* specie*s* also have the potential to generate biofuels (hydrogen) [11], biopolymers (polyhydroxyalkanoate) [12], and bioactive molecules (acyl-homoserine lactonases) [13, 14]. Therefore, *Bacillus* is important not only in traditional territories like agriculture, medical treatment, and pharmaceutical manufacturing but also contributes to some emerging territories such as sustainable and clean energy in the future.

Bacteriophages (phages) are present in all environments in which bacteria survive, with genetic diversity, and play an important role in the evolution of bacterial host cells [15, 16]. The main mediator of phage evolution is horizontal gene transfer (HGT) between different ancestors, which accounts for the diversity and uniqueness of phages [17]. Lytic phages act as bacterial killers lysing host cells, influencing the ecology and evolution of bacterial populations by affecting the number of bacterial populations in different environments, selecting resistant types with potentially altered phenotypes, and changing the competitive hierarchy of bacterial communities [18–22]. The whole genome sequences of bacteria revealed an abundance of lysogenic phage sequences in the genomes of many bacterial species [23]. Interestingly, some phage genes originated in bacterial cells, and these phage-introduced genes (called auxiliary metabolic genes) in host cells can modulate host cell metabolism during infection [24–26]. Therefore, phages have served as vectors of horizontal gene transfer and drivers of bacterial evolution.

Although *Bacillus* has been widely used for various purposes, little is known about *Bacillus* phages. Fewer studies have been conducted on the different functional fragments carried by *Bacillus* phages, the linkage between the genomes of different *Bacillus* phages, and whether additions or deletions of gene fragments occurred during the evolution of *Bacillus* phages and whether additions or deletions had an effect on *Bacillus* phages. Therefore, in this work, we took some representative *Bacillus* prophages and lytic phages as the subjects, characterized the genomes of phages to explore their evolutionary relationships, and analyzed the information of proteins encoded by *Bacillus* phages. Our results showed the following, (1) *Bacillus* phages carried different functional fragments that might have different effects on the host *Bacillus* species. (2) A clear genomic boundary existed between *Bacillus* prophages and lytic phages. (3) *Bacillus* phages underwent the evolutionary process of gene mutations, sequence losses, duplications and host switching, resulting in low similarity between *Bacillus* phage genomes. (4) The lysis module plays an important role in the evolution of *Bacillus* phage. Our work reveals the biological functions, genomic features and evolutionary relationships of phages, laying the foundation for a better understanding of key questions in microbial ecology, evolution and potential biotechnological applications.

## Results

### Bioinformatic Analysis of Proteins Encoded by *Bacillus* phages

**Fig. 1:**
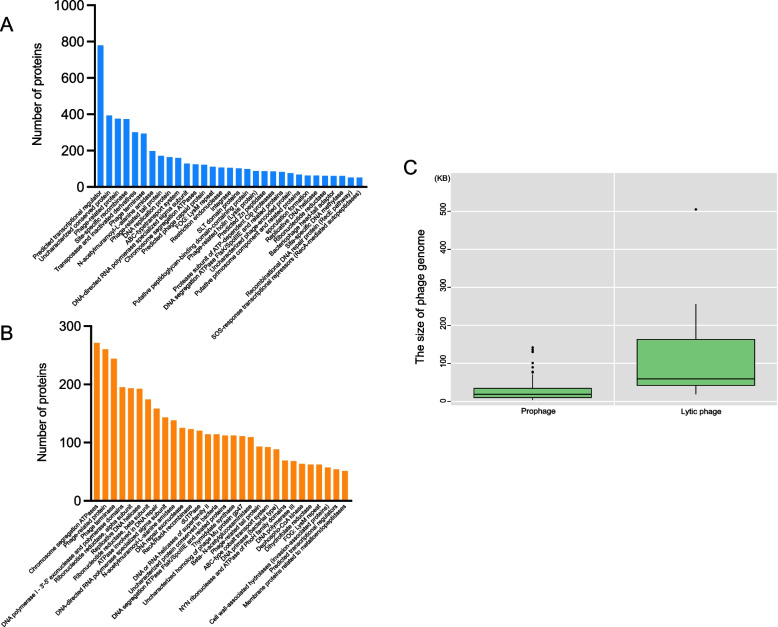
The information of *Bacillus* phage sequences and genomes including functional classification of proteins encoded by *Bacillus* phage and the size of *Bacillus* phage sequences and genomes. **A** The proteins related to phage lifecycles were encoded by 619 predicted prophages sequences. Only proteins with more than 50 homologs of the same function were shown. **B** The proteins related to phage lifecycles were encoded by 236 lytic phages genomes. Only proteins with more than 50 homologs of the same function were shown. **C** The Box-plot of *Bacillus* phage sequence and genome size, including the 619 prophage sequences and the 236 lytic phage genomes

In this article, nucleotide sequences of 619 prophages (Table S1) predicted by 178 *Bacillus* genomes and 236 lytic phages were used to compare the sequences and genome size, annotate protein function, and further statistical analysis (Fig. 1). The length of *Bacillus* prophage sequences ranged from 4 – 142 kb, averaging about 24 kb, and the length of *Bacillus* lytic phage genomes ranged from 19 – 590 kb, averaging about 99 kb (Fig. 1C). The genomes size of *Bacillus* lytic phage was nearly four times larger than the sequences of *Bacillus* prophage. The results of the functional classification of proteins encoded by all *Bacillus* phages were as follows: For *Bacillus* prophages, a total of 8457 proteins were predicted and identified as 894 kinds of proteins were homologous with proteins in the COG database. As shown in Fig. 1A, lots of *Bacillus* prophage proteins were associated with the life cycle of phages, such as phage capsid protein, tail proteins, related structural proteins, phage genome integration-related proteins, DNA replication and repair related proteins, phage infection-related proteins, lytic and lysogenic regulation proteins, etc. In addition, abundant predicted transcriptional regulators were also included. For *Bacillus* lytic phages, 5889 proteins were predicted and identified as 335 kinds of proteins were homologous with protein in the COG database. Functional analysis of *Bacillus* lytic phage proteins revealed that most were phage structural proteins, phage infection-related, DNA synthesis, and replication-related proteins (Fig. 1B). *Bacillus* prophage and *Bacillus* lytic phages still had many genes encoding unknown functional proteins in their genomes. Notably, proteins of interest were identified in these *Bacillus* phage genomes, such as proteins related to spore formation, proteins associated with cell wall biosynthesis, proteins related to cell wall-associated hydrolases (invasion-associated proteins), and proteins about exopolysaccharide biosynthesis (Table S2). These proteins also might, directly or indirectly, take part in phage-host interactions. In addition, some *Bacillus* phages were also found to carry virulence factors, including Hemolytic enterotoxin [27], Phospholipase C [28], Metalloprotease [29], Chitinase [30], etc., which might lead to transmission of virulence factors through horizontal gene transfer (Table S2).

### The Similarity Analysis *Bacillus* Phage Genomes

**Fig. 2:**
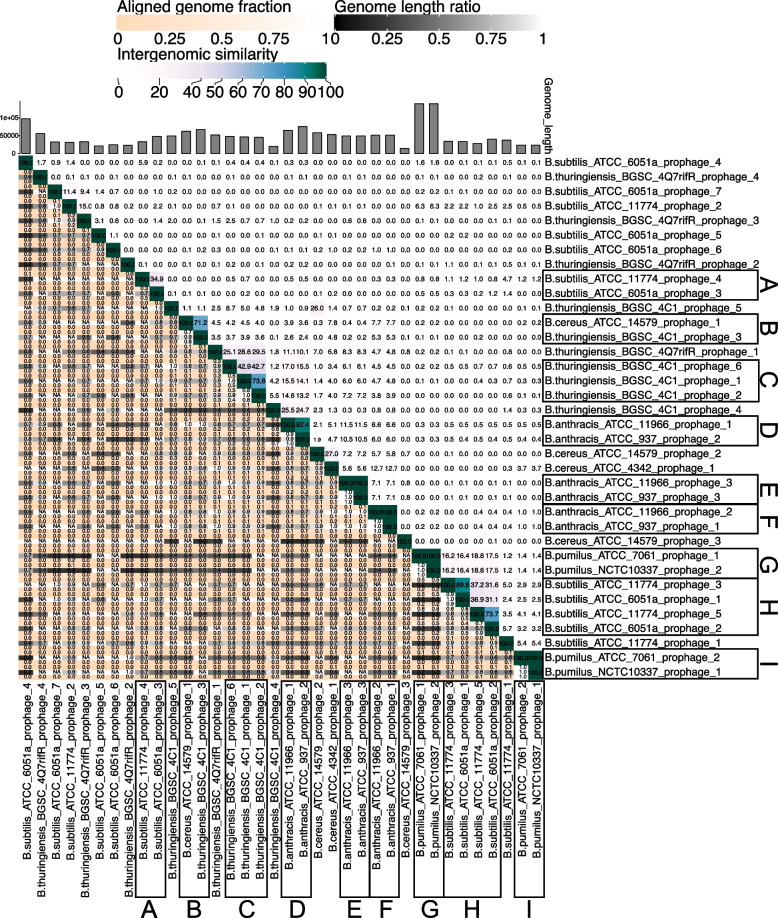
Heat map of 36 *Bacillus* prophage complete genome sequences. Prophages with similar genomes in the same frame, and each frame was marked with an alphabet

**Fig. 3:**
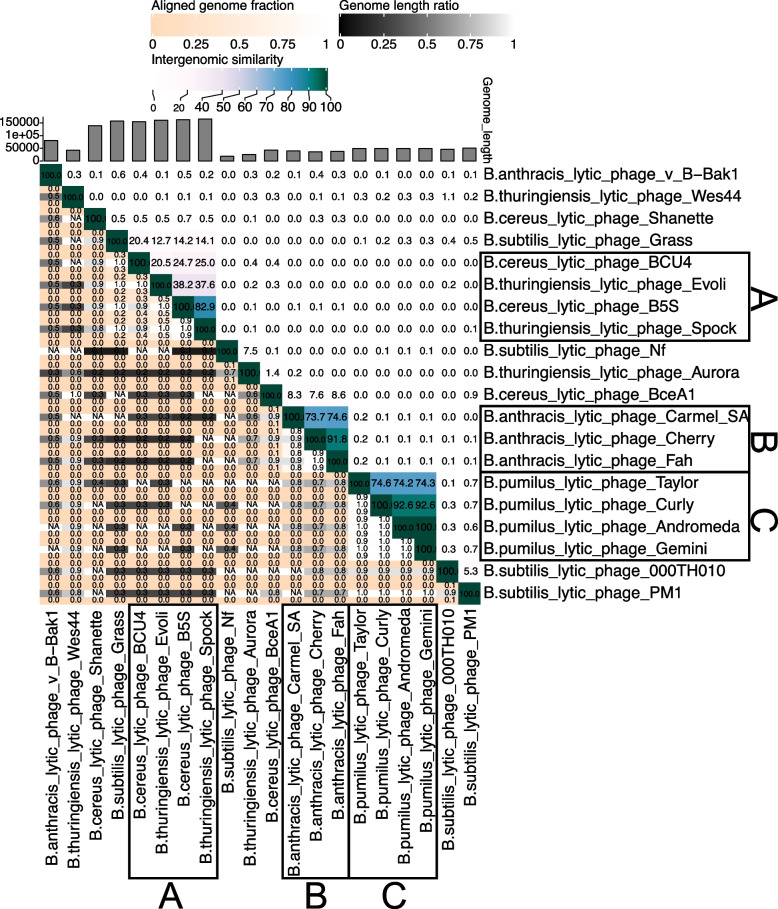
Heat map of 20 *Bacillus* lytic phage complete genome sequences. Lytic phages with similar genomes in the same frame, and each frame was marked with an alphabet

**Fig. 4:**
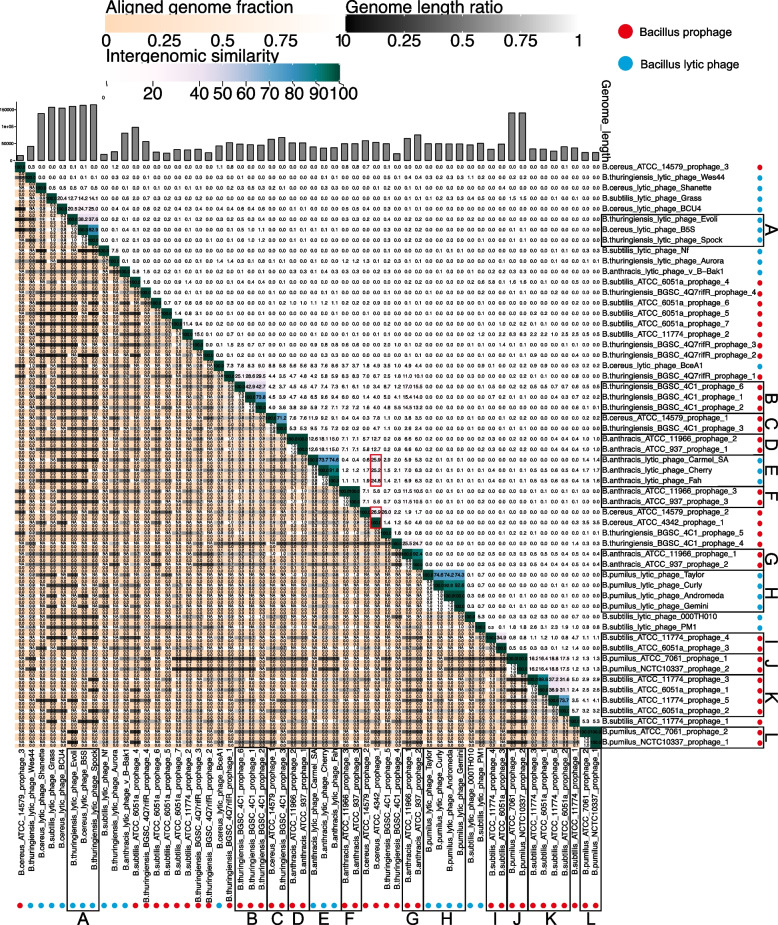
Heat map of 36 *Bacillus* prophage and 20 *Bacillus* lytic phage complete genome sequences. Phages with similar genomes in the same frame, and each frame was marked with an alphabet

Sequences of 36 predicted prophages and 20 lytic phages were used for the evolutionary analysis of *Bacillus* phage (Tables S3 and S4). Figures 2, 3 and 4 were heat maps consisting of the whole genome of *Bacillus* phage. From the figure, we observed that most phage genomes had a low similarity, while some phage genomes were clustered together with high similarity for lysogenic and lytic phages (Figs. 2 and 3). Some phages with similar genomes whose hosts belong to the same *Bacillus* species are frame D, frame E, frame F, frame G, frame H, and frame I in Fig. 2, as well as frame B and frame C in Fig. 3. Another part of phages with similar genomes whose hosts belong to two different species of *Bacillus*, *B. cereus* and *B. thuringiensis*, respectively, are frame B in Fig. 2 and frame A in Fig. 3. Both *B. cereus* and *B. thuringiensis* belong to the *B. cereus* bacterial group. Their genetic similarity is extremely high except for the plasmid gene, which should be considered the same species [31]. In addition, frame A in Fig. 3 was worthy of our attention. It is composed of five virulent phages, namely *B. subtilis* lytic phage Grass, *B. cereus* lytic phage BCU4, *B. thuringiensis* lytic phage Evoli, *B. cereus* lytic phage B5S and *B. thuringiensis* lytic phage Spock. The genome similarity decreased from *B. thuringiensis* lytic phage Spock to *B. subtilis* lytic phage Grass. This may be a *Bacillus* phage genome evolutionary process, from singular to diverse and from one species to more species. Figure 4, which consisted of the whole genomes of prophages and lytic phages, showed that the genomes of almost no prophages were similar to those of lytic phages. In summary, from the perspective of phage genome similarity, the genomes of *Bacillus* phages are diverse, and the infestation range of *Bacillus* phages is limited to *Bacillus* that are of the same species. Phages that infected different *Bacillus* species showed little genomic similarity, and even phages that infected the same *Bacillus* species showed considerable genomic differences. Furthermore, a boundary existed between *Bacillus* prophages and lytic phages. Significant differences were found not only in genome size but also in genome similarity. The genomes size of *Bacillus* lytic phage was nearly four times larger than the sequences of *Bacillus* prophage, with little similarity between *Bacillus* prophage sequences and lytic phage genomes.

Interestingly, *B. anthracis* lytic phages Carmel SA, *B. anthracis* lytic phages Cherry, *B. anthracis* lytic phages Fah, *B. cereus* ATCC 14579 prophage 2 and *B. cereus* ATCC 4342 prophage 1 appear to be somewhat related (marked with red frame) (Fig. 4). Of these five *Bacillus* phages, the genomic similarity of the three lytic phages was over 74.6%, and they showed about 25% genomic similarity to *B. cereus* ATCC 4342 prophage 1. Also, the genomic similarity between *B. cereus* ATCC 4342 prophage 1 and *B. cereus* ATCC 14579 prophage 2 was about 25%.

### Comparative Analysis of Similar *Bacillus* Phages Genome

**Fig. 5:**
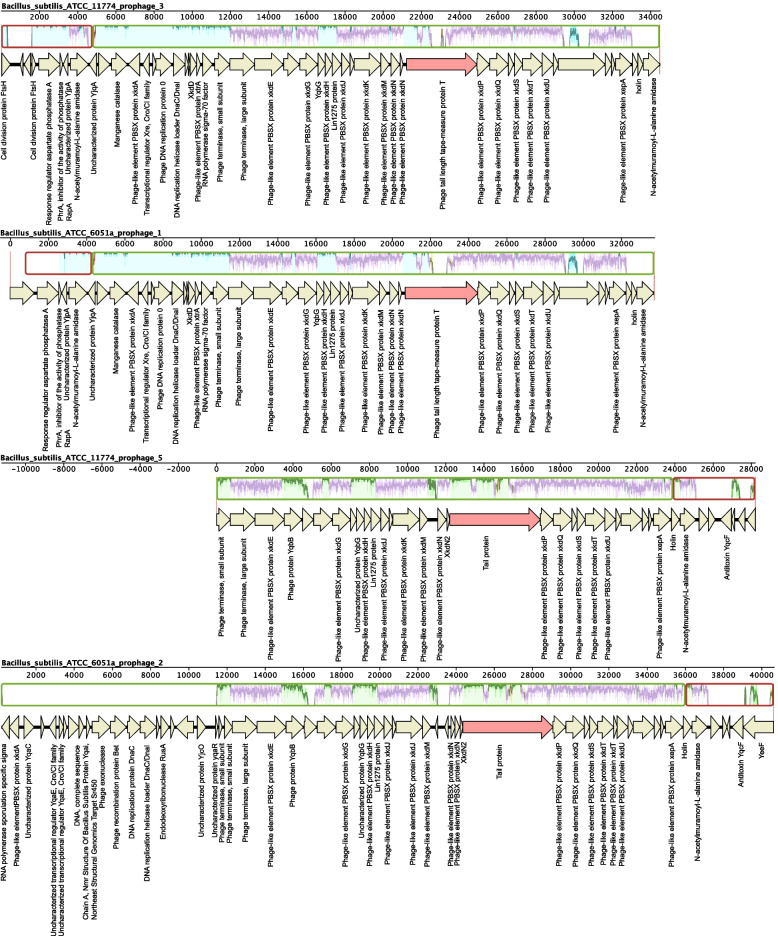
The genomes mauve alignment of 4 similar *Bacillus* prophage sequences. MAUVE alignments showing the conserved structure between the similar *Bacillus* phage genomes. Locally Collinear Blocks (LCB) are indicated by corresponding colored region. The lavender color represents the conserved regions of all genomes. The green and yellow color represents the conserved regions between two genomes. The red color represents the conserved regions between three phages genomes. Annotations are reported by the arrow below the LCBs

**Fig. 6:**
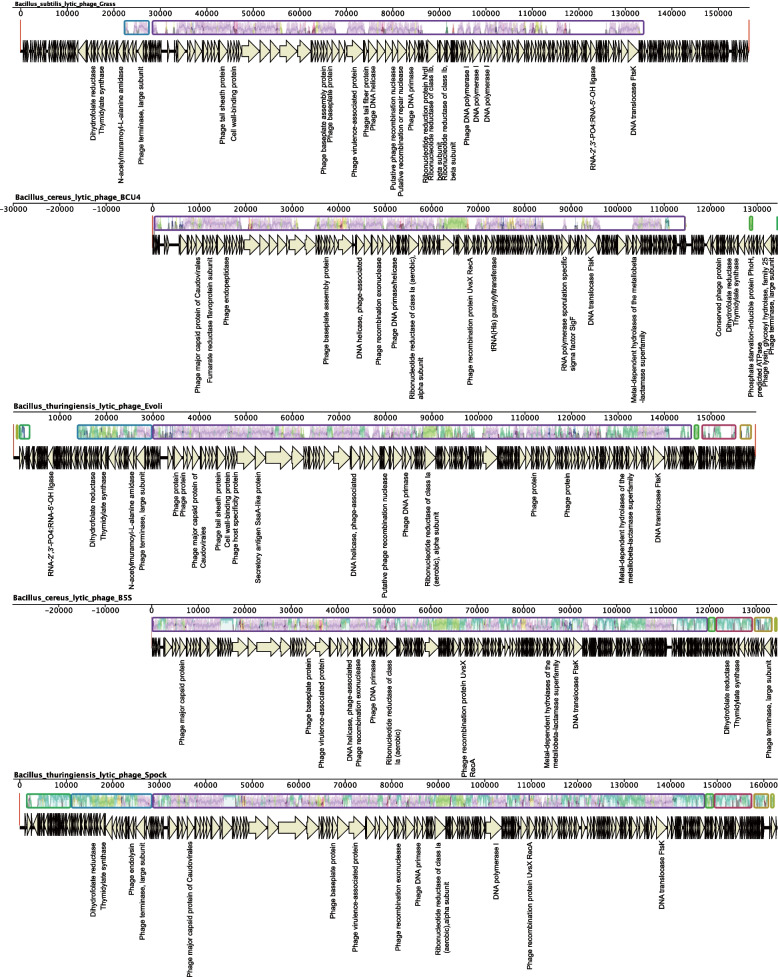
The genomes mauve alignment of 5 similar *Bacillus* lytic phage genomes. Whole genome MAUVE alignments showing the conserved structure between the similar *Bacillus* phage genomes. Locally Collinear Blocks (LCB) are indicated by corresponding colored region. The lavender color represents the conserved regions of all genomes. The dark purple, pink and blue colors represent the conserved regions between the different phage genomes. Annotations are reported by the arrow below the LCBs

A group of prophages (Fig. 2, frame H) and a group of lytic phages (Fig. 3, frame A) were selected for comparative genomic analysis to explore the evolution and laws of phages. In comparison to the genomes of the *Bacillus* prophages, shown in Fig. 5, four prophages have 25 shared homologous proteins. Nine proteins had clear functions, including capsid portal protein, tail tube protein, tail assembly chaperone protein, base plate assembly protein, terminase large subunit, terminase small subunit, N-acetylmuramoyl-L-alanine amidase, and Lin1275 protein (putative tail-component). All other proteins were putative or hypothetical proteins. And five lytic phage genomes have 69 shared homologous proteins. Twelve proteins had clear functions, including phage protein, phage major capsid protein, terminase large subunit, thymidylate synthase, phage DNA primase, DNA translocase FtsK and ribonucleotide reductase (Fig. 6). The genomes mauve alignment showed that the genes encoding these proteins were homologous. In the *Bacillus* prophage sequences, both homologous (lavender region) and non-homologous (green parts) fragments existed as whole large segments; this phenomenon might result from genetic recombination (Fig. 5). Compared to the prophage, homologous and non-conservative regions in the lytic phage genome were more random (Fig. 6). Notably, conservative segments were located in the middle of the genomes, and the non-conservative segments were mainly located at both ends of the sequence in the *Bacillus* prophage sequences (Fig. 5). The results showed that different *Bacillus* phages evolve in different regularity during evolution. The *Bacillus* prophage mainly evolved by recombining the genome, and the recombination occurred at both ends of the genome. The *Bacillus* lytic phages evolved through genetic mutations randomly during evolution. Interestingly, the conservative regions of all four prophage sequences had a non-homologous gene segment (marked with light red arrows), they were both the genes that encoded the phage tail protein (Fig. 5). The previous study showed that during co-evolution with host bacteria, some *Bacillus* phages developed the ability to infect resistant strains [32]. Mutational evolution of phage tail proteins is probably one of the strategies.

**Fig. 7:**
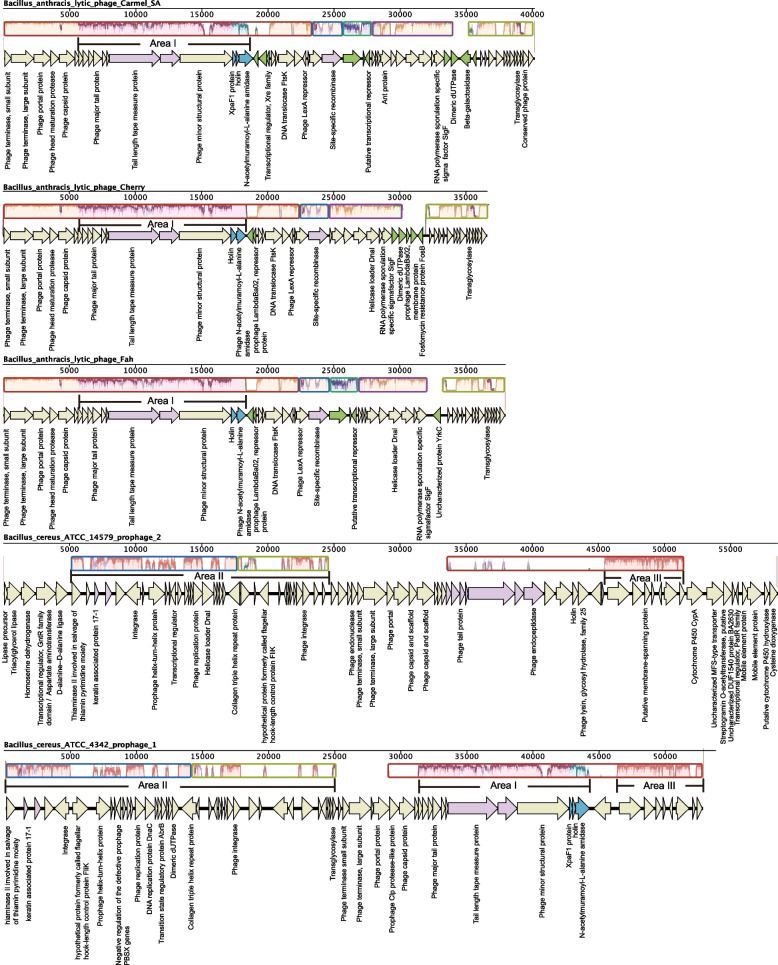
The genomes mauve alignment of 5 similar *Bacillus* phage genomes including 3 lytic phage genomes and 2 *Bacillus* prophage sequences. The genomes mauve alignment of 5 similar *Bacillus* lytic phage genomes. Whole genome MAUVE alignments showing the conserved structure between the similar *Bacillus* phage genomes. Locally Collinear Blocks (LCB) are indicated by corresponding colored region. The lavender color represents the conserved regions of all genomes. Annotations are reported by the arrow below the LCBs

The five interesting *Bacillus* phages in Fig. 4 were also selected for genomic covariance analysis using Mauve, and the results are shown in Fig. 7. Compared to the high similarity between these three lytic phage genomes, these five Bacillus genomic sequences had few homologous sequences, but some traces of homology were still present. As in Fig. 7, several discontinuous mauve homologous fragments were present in the area marked by the mauve arrow. In addition to the above homologous fragments, a homologous sequence (AreaI) was found between the genome of ATCC 4342 prophage 1, Carmel SA, Cherry, and Fah. Two homologous sequences (AreaII and Area III) were also observed between the genome of ATCC 4342 prophage 1 and ATCC 14579 prophage 2. In contrast, no additional homologous sequences occurred between the genome of ATCC 14579 prophage 2, Carmel SA, Cherry, and Fah. Taken together, the five phages may have evolved from the one phage. The phage genome was likely similar to the three lytic phages, and due to some factors, the genome first becomes the ATCC 4342 prophage 1 genome and subsequently changes from the ATCC 4342 prophage 1 genome to the ATCC 14579 prophage 2 genome. So far, the ATCC 14579 prophage 2 genome has evolved completely differently from its ancestor.

**Fig. 8:**
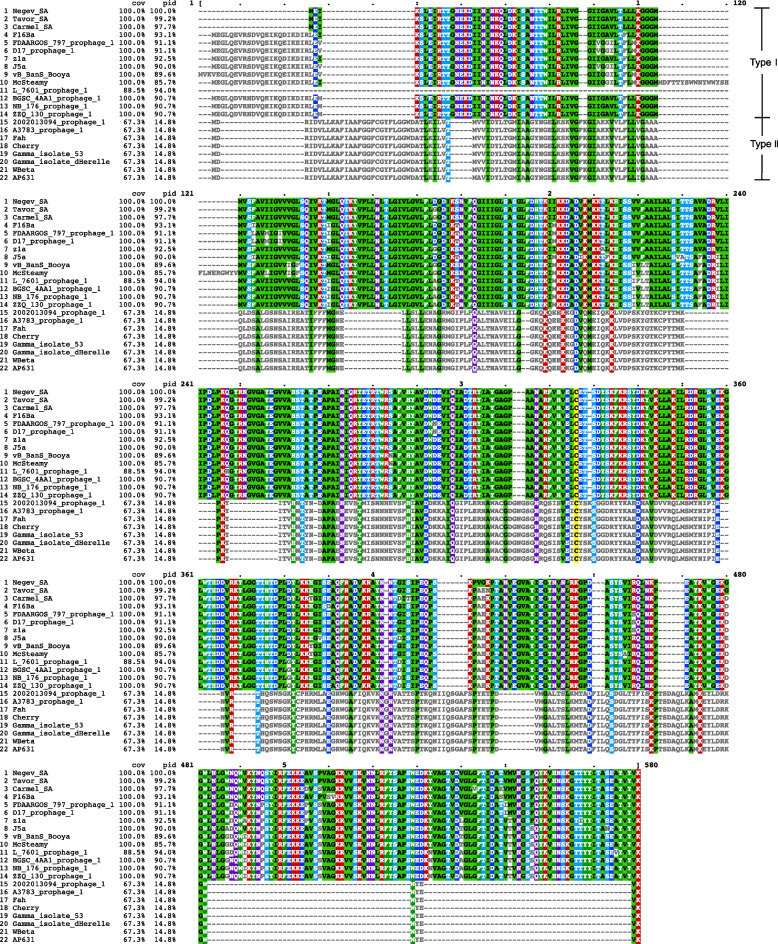
Protein sequence alignment of 22 lysis modules from *Bacillus* phage genome. According to the sequence similarity, the 22 lysis modules were divided into two types, Type I and Type II

The areas corresponding to the blue arrows were gene fragments encoding the holin family (XpaF1 and holin) and endolysin proteins (N-acetylmuramoyl-L-alanine amidase), which we referred to as the lysis module. Both were located behind the genes encoding the phage minor structural protein in their respective genomes. The lysis modules showed low similarity between the three lytic phage genomes, although the preceding and following sequences of the lysis module were all highly homologous. As described above, we searched for genome fragments similar to the three lytic phage genomes from NCBI and the results were shown in Table S5. Then, we searched for the lysis module where the gene fragments encoding the Holin family and endolysin proteins were adjacent form from these genome fragments, and downloaded all the lysis module protein sequences. These protein sequences were used for comparative analysis, and the results are shown in Fig. 8, the protein sequences marked with the same number and name are concatenated. These sequences were divided into two clusters, which indicated that these lysis modules were classified into two types. Further analysis of the sequences of the two types of proteins revealed that their similarity was only about 15%. Here, we named them type I and type II, represented by Carmel SA and Cherry, respectively. Interestingly, all homologous prophages from other species of *Bacillus* had the type I lysis module (Table S5). Subsequently, homologous sequences of both types of lysis modules were searched in NCBI, and the results are shown in Tables S6 and S7. In addition to those lytic phages and *Bacillus* in Table S5, the type I lysis module was also found in the genomes of some *B. thuringiensis*, *B. cereus*, and other *Bacillus* species. In contrast, the type II lysis module was only found in the genomes of some *B. anthracis*. This result coincided with the result in Table S5. In summary, we conclude that the two types of lysis modules of *B. anthracis* phages are related to the species of the hosts they infect.

A few additional areas of difference (marked by green and blue arrows) deserved our attention. First were the gene sequences corresponding to the green arrows, which were present in the genome of only one or two of the three lytic phages. For example, the gene sequence encoding a putative transcriptional repressor (ACLAME 12) was only present in the genomes of Carmel SA and Fah, while it was missing from the genome of Cherry. In addition, the genomes of these three cell-type phages had an un-stable region at the same corresponding position. Several non-homologous genes encoding different proteins were present in the region (Fig. 7). Such as beta-galactosidase, dimeric dUTPase, Fosfomycin resistance protein FosB, LambdaBa02 membrane protein, and some unknown function proteins. Several functionally annotated gene sequences were used to search for homologous sequences in NCBI, and the results are shown in Table S8. These genes-encode proteins involved in lactose metabolism, antibiotic resistance, regulatory factors, membrane proteins, and homologous sequences of these genes were found in different *Bacillus* bacteria. This phenomenon is likely caused by the horizontal gene transfer at the phage level.

## Discussion

In this paper, through bioinformatic analysis of the *Bacillus* phage genomes, we found that in addition to the essential proteins required for phage survival, *Bacillus* phage encoded a number of related proteins involved in host growth or metabolic activities. These included a number of transcription factors, sporulation, and cell wall synthesis-related proteins (Table S2). These proteins were probably directly or indirectly involved in phage-host interactions. As well as the large number of unknown functional proteins encoded by *Bacillus* phages would become an essential booster to drive the co-evolution of *Bacillus* phages with their hosts. In addition, we found that some *Bacillus* phages carry virulence factors (Table S2). These virulence factors may be transferred between different *Bacillus* strains by means of horizontal gene transfer by phages that act as mobile elements, allowing non-virulent bacteria to become pathogenic. Previous studies that have demonstrated that the pathogens *B. anthracis*, *B. cereus*, and *B. thuringiensis* were commonly infected by arbitrium-carrying mobile elements, which often carried toxins essential for pathogenicity [33]. Therefore, we should pay more attention to the fact that *Bacillus* phages might transfer pathogenic gene fragments to make the non-toxic bacteria pathogenic.

As a kind of simple biological entity on the planet, phages own relatively small genomes. Still, they show impressive genomic diversity and complex evolutionary relationships, which is also true for *Bacillus* phages. In this study, some representative *Bacillus* phage genomes were used to construct the heat maps, and the results indicated that *Bacillus* phages showed remarkable diversity at the nucleotide sequence level (Figs. 2 and 3). Furthermore, a boundary existed between *Bacillus* prophages and lytic phages in the genome. Significant differences were found in genome similarity, size, and evolutionary mechanisms (Figs. 1C, 5, and 6). From an evolutionary perspective, phages evolved in response to survival pressures in favor of infecting or co-evolving with their hosts [17]. For example, mutations in genes encode phage tail proteins (Fig. 5) facilitate phage infection of hosts and may also enable cross-species infection [32]. Our study also identified a group of five *Bacillus* phages which probably evolved from one phage (Fig. 7). In the complicated evolutionary process, these phage genomes were likely to be driven by different mechanisms. For instance, genetic mutations, sequence losses, transfer, and host switching have resulted in genetic diversity and low genome similarity between phages.

In addition, two different types of lysis modules, type I and type II were found in the five phages. Further analysis revealed some connection between the types of the two phage lysis modules and the host range of the phages, and the phage possessing the lysis module of type I could infect other species of *Bacillus* more often than the phage possessing type II (Table S5, Fig. 8, Tables S6 and S7). The lysis modules include gene fragments encoding the holin family (XpaF1 and holin) and endolysin proteins (N-acetylmuramoyl-L-alanine amidase). It was found that the lysis module plays a role in the recognition of host bacterial substrates [34, 35]. During the evolutionary process, the primal *B. anthracis* phage lysis module gene was mutated into the present type I lysis module, which let the phage get an ability to recognize more other species of *Bacillus* and then infect them. This phenomenon has extended the host range of *Bacillus* phages, thus promoting the evolution and diversity of *Bacillus* phages.

## Conclusions

The research on *Bacillus* phages is important since *Bacillus* is widely used in agricultural and industrial productions. However, the phylogeny of *Bacillus* phages remains a knowledge gap in *Bacillus* studies. In summary, we analyzed the complete genomes of *Bacillus* prophages and lytic phages and systematically described their protein function, diversity, and genome evolution. This study contributes to understanding the *Bacillus* phage genomic characteristics, *Bacillus* phage-host interactions, and the evolutionary relationships of *Bacillus* phages.

## Materials and methods

### Collection of *Bacillus* phage genomes

In NCBI, strains of 178 *Bacillus* species have been sequenced by others, as we selected and downloaded the whole genome sequences of 178 strains from all the *Bacillus* species (one strain per species) for prophage prediction using PHASTER software (Table S1). Meanwhile, 236 strains of *Bacillus* lytic phage genomes sequences were downloaded from NCBI (Table S9). The predicted *Bacillus* prophage sequences and the *Bacillus* lytic phage genomes obtained from NCBI were used to annotate and analyze functional genes. All the genome sequence data were from NCBI before 30th December 2022 (https://www.ncbi.nlm.nih.gov/nuccore).

The more familiar *Bacillus* species that have been under study are *Bacillus anthracis*, *Bacillus cereus*, *Bacillus thuringiensis*, *Bacillus subtilis*, and *Bacillus pumilus*. Most of the *Bacillus* lytic phages with the whole genome published in the NCBI database were isolated from them. Therefore, the five *Bacillus* phages mentioned above were chosen as representatives of the *Bacillus* phages for analysis. The genome sequences of 20 *Bacillus* lytic phages and 36 *Bacillus* prophages were selected for the evolutionary analysis of the *Bacillus* phage, and their hosts were all the five *Bacillus* mentioned above. The *Bacillus* prophage nucleotide sequences were obtained by prediction using PHASTER. Ten *Bacillus* genomes (two per species, three *B. pumilus*) were downloaded from NCBI to predict *Bacillus* prophage nucleotide sequences (Table S3). From these prophage sequences, intact or fragment sizes of more than 20 kb were selected, a total of 36 *Bacillus* prophage sequences (Table S3). The *Bacillus* lytic phage genomes were obtained from NCBI (four per species) total of 20 (Table S4).

### Prediction of *Bacillus* prophage nucleotide sequences

All *Bacillus* prophage nucleotide sequences were predicted using PHASTER (http://phaster.ca/). PHASTER is a tool for identifying prophage sequences, including phage sequence identification, protein identification, and evaluating the completeness of the putative prophage. Based on the completeness of the predicted phage sequences, the prophages were classified into intact prophage, questionable prophage, and incomplete prophage.

### Annotations and analysis of the *Bacillus* phage genome

The proteins encoded by the prophage and lytic phage sequences were predicted by GeneMark web software (http://opal.biology.gatech.edu/GeneMark) [36]. The functional annotation and the COG (Cluster of Orthologous Groups of proteins) classification of the proteins was performed using WebMGA (http://weizhong-lab.ucsd.edu/webMGA/). WebMGA is a customizable web server for fast metagenomic analysis. The bar chart was drawn by graphpad prism 9.5.1, and the Box plot was drawn by the OmicShare tools, an online platform for data analysis (https://www.omicshare.com/tools).

### The evolutionary analysis of the *Bacillus* phage

The heat maps were made with all the phage sequences in VIRIDIC web (http://rhea.icbm.uni-oldenburg.de/VIRIDIC/). VIRIDIC was developed in R 3.5 programming language and was a new tool for calculating virus intergenomic similarities. It uses the traditional algorithm, which is also used by the Bacterial and Archaeal Viruses Subcommittee and the International Committee on Taxonomy of Viruses (ICTV) [37]. Evolutionary analysis of phage genomes was performed using the Rast (https://rast.nmpdr.org), Mauve 2.3.1and Easyfig 2.2.5. Firstly, the fasta format nucleic acid sequence files were annotated into GBK format files by Rast. Then the files in GBK format were then imported into Easyfig for protein visualization and Mauve for homology analysis. Next, the protein visualization result figure of Easyfig (protein arrows) and the analysis result figure of Mauve were integrated in one figure. Finally, the analysis was performed based on the homology results of Mauve. Rast is an automated annotation website for complete, or near-complete, archaeal and bacterial genomes [38].Mauve is a system for efficiently constructing multiple genome alignments in the presence of large-scale evolutionary events such as rearrangement and inversion [39]. Easyfig is an application for creating linear comparison figures of multiple genomic loci. BLAST comparisons between multiple genomic regions can be generated, ranging from single genes to whole prokaryote chromosomes. Protein sequence alignment of the lysis module was performed by MAFFT (https://mafft.cbrc.jp/alignment/server/) and MView (https://www.ebi.ac.uk/Tools/msa/mview/). First, the protein sequences of the lysis module were downloaded from NCBI. Then compared them in MAFFT and used MView to make the figure. MAFFT is an online service for multiple sequence alignmen. MView is a tool for converting the results of a sequence database search into colored multiple alignments of hits stacked against the query.

## Supplementary Information

**Additional file 1: Table S1.** The number of predicted prophages in 178 *Bacillus* strains.

**Additional file 2: Table S2.** A portion of functional proteins carried by *Bacillus* phage.

**Additional file 3: Table S3.** The information of the 36 *Bacillus* prophages and their host.

**Additional file 4: Table S4.** The information of the 20 lytic phages.

**Additional file 5: Table S5.** The homologous sequences of *Bacillus* lytic phages Carmel_SA, Cherry and Fah were searched in NCBI.

**Additional file 6: Table S6.** Description of homologous sequences of the type I lysis module.

**Additional file 7: Table S7.** Description of homologous sequences of the type II lysis module.

**Additional file 8: Table S8.** The blast result of some several functionally annotated gene sequences from NCBI.

**Additional file 9: Table S9.** The information of  236 *Bacillus* lytic pahge.

## Data Availability

All the genome sequences were downloaded from NCBI (https://www.ncbi.nlm.nih.gov/nuccore). Among them, the genomes of *Bacillus* prophages were predicted by software, so we provided the information of their hosts. And the genome of *Bacillus* lytic phage was obtained from NCBI download. All details (including accession numbers) are available in the supplementary information files.

## References

[1] Turnbull PCB, Kramer JM, Melling J (1991). *Bacillus*. Manual Clin Microbiol.

[2] Bressuire-Isoard C, Broussolle V, Carlin F (2018). Sporulation environment influences spore properties in *Bacillus*: evidence and insights on underlying molecular and physiological mechanisms. FEMS Microbiol Rev.

[3] Kovacs AT (2019). Bacillus subtilis. Trends Microbiol.

[4] Penha RO, Vandenberghe LPS, Faulds C, Soccol VT, Soccol CR. *Bacillus* lipopeptides as powerful pest control agents for a more sustainable and healthy agriculture: recent studies and innovations. Planta. 2020;251(3); 10.1007/s00425-020-03357-7. 10.1007/s00425-020-03357-7 32086615

[5] Rabbee MF, Baek KH. Antimicrobial Activities of Lipopeptides and Polyketides of *Bacillus velezensis* for Agricultural Applications. Molecules. 2020;25(21); 10.3390/molecules25214973. 10.3390/molecules25214973 PMC7662345 33121115

[6] Nazari M, Smith DL. A PGPR-Produced Bacteriocin for Sustainable Agriculture: a Review of Thuricin 17 Characteristics and Applications. Front Plant Sci. 2020;11; 10.3389/fpls.2020.00916. 10.3389/fpls.2020.00916 PMC7358586 32733506

[7] Nithyapriya S, Lalitha S, Sayyed RZ, Reddy MS, Dailin DJ, El Enshasy HA, et al. Production, Purification, and Characterization of Bacillibactin Siderophore of *Bacillus subtilis* and Its Application for Improvement in Plant Growth and Oil Content in Sesame. Sustainability. 2021;13(10); 10.3390/su13105394.

[8] Ohba M, Mizuki E, Uemori A (2009). Parasporin, a New Anticancer Protein Group from *Bacillus thuringiensis*. Anticancer Res.

[9] Melo ALD, Soccol VT, Soccol CR (2016). *Bacillus thuringiensis*: mechanism of action, resistance, and new applications: a review. Crit Rev Biotechnol.

[10] Shafi J, Tian H, Ji MS (2017). *Bacillus* species as versatile weapons for plant pathogens: a review. Biotechnol Biotechnol Equip.

[11] Magrini FE, Castilhos A, Lora LB, Paesi S. Strategies of co-cultures and bioaugmentation by *Bacillus amyloliquefaciens*, *Clostridium bifermentans*, *Enterobacter muelleri*, and *E. tabaci* for increasing the production of hydrogen from raw glycerol. Biomass Bioenergy. 2023;168; 10.1016/j.biombioe.2022.106672.

[12] Israni N, Venkatachalam P, Gajaraj B, Varalakshmi KN, Shivakumar S. Whey valorization for sustainable polyhydroxyalkanoate production by *Bacillus megaterium*: Production, characterization and in vitro biocompatibility evaluation. J Environ Manag. 2020;255; 10.1016/j.jenvman.2019.109884. 10.1016/j.jenvman.2019.109884 32063322

[13] Noor AO, Almasri DM, Basyony AF, Albohy A, Almutairi LS, Alhammadi SS, et al. Biodiversity of N-acyl homoserine lactonase (aiiA) gene from *Bacillus subtilis*. Microb Pathogenesis. 2022;166; 10.1016/j.micpath.2022.105543. 10.1016/j.micpath.2022.105543 35460864

[14] Kumar P, Patel SKS, Lee JK, Kalia VC (2013). Extending the limits of *Bacillus* for novel biotechnological applications. Biotechnol Adv.

[15] Stone E, Campbell K, Grant I, McAuliffe O. Understanding and Exploiting Phage-Host Interactions. Viruses-Basel. 2019;11(6); 10.3390/v11060567. 10.3390/v11060567 PMC6630733 31216787

[16] Oechslin F, Zhu XJ, Dion MB, Shi R, Moineau S. Phage endolysins are adapted to specific hosts and are evolutionarily dynamic. Plos Biology. 2022;20(8); 10.1371/journal.pbio.3001740. 10.1371/journal.pbio.3001740 PMC9371310 35913996

[17] Dion MB, Oechslin F, Moineau S (2020). Phage diversity, genomics and phylogeny. Nat Rev Microbiol.

[18] Koskella B, Taylor TB (2018). Multifaceted Impacts of Bacteriophages in the Plant Microbiome. Annual Rev Phytopathol..

[19] Zuppi M, Hendrickson HL, O'Sullivan JM, Vatanen T. Phages in the Gut Ecosystem. Front Cell Infect Microbiol. 2022;11; 10.3389/fcimb.2021.822562. 10.3389/fcimb.2021.822562 PMC8764184 35059329

[20] Tuttle MJ, Buchan A (2020). Lysogeny in the oceans: Lessons from cultivated model systems and a reanalysis of its prevalence. Environ Microbiol.

[21] Cazares D, Cazares A, Figueroa W, Guarneros G, Edwards RA, Vinuesa P. A Novel Group of Promiscuous Podophages Infecting Diverse Gammaproteobacteria from River Communities Exhibits Dynamic Intergenus Host Adaptation. Msystems. 2021;6(1); 10.1128/mSystems.00773-20. 10.1128/mSystems.00773-20 PMC7857530 33531404

[22] Wu HQ, Wan SC, Ruan CJ, Niu XY, Chen GW, Liu Y, et al. Phage-bacterium interactions and nutrient availability can shape C and N retention in microbial biomass. Eur J Soil Sci. 2022;73(4); 10.1111/ejss.13296.

[23] Casjens S (2003). Prophages and bacterial genomics: what have we learned so far?. Mol Microbiol.

[24] Nadeem A, Wahl LM (2017). Prophage as a genetic reservoir: Promoting diversity and driving innovation in the host community. Evolution.

[25] Kropinski AM, Turner D, Nash JHE, Ackermann HW, Lingohr EJ, Warren RA, et al. The Sequence of Two Bacteriophages with Hypermodified Bases Reveals Novel Phage-Host Interactions. Viruses-Basel. 2018;10(5); 10.3390/v10050217. 10.3390/v10050217 PMC5977210 29695085

[26] Bhambhani A, Iadicicco I, Lee J, Ahmed S, Belfatto M, Held D (2020). Bacteriophage SP01 Gene Product 56 Inhibits *Bacillus subtilis* Cell Division by Interacting with FtsL and Disrupting Pbp2B and FtsW Recruitment. J Bacteriol.

[27] Rohmer C, Wolz C (2021). The Role of hlb-Converting Bacteriophages in *Staphylococcus aureus* Host Adaption. Microbial Physiol.

[28] Diene SM, Corvaglia AR, Francois P, van der Mee-Marquet N, Regional Infection Control Grp C. Prophages and adaptation of Staphylococcus aureus ST398 to the human clinic. Bmc Genomics. 2017;18; 10.1186/s12864-017-3516-x. 10.1186/s12864-017-3516-x PMC5294865 28166723

[29] Qumar S, Majid M, Kumar N, Tiwari SK, Semmler T, Devi S, et al. Genome Dynamics and Molecular Infection Epidemiology of Multidrug-Resistant Helicobacter pullorum Isolates Obtained from Broiler and Free-Range Chickens in India. Applied and Environmental Microbiology. 2017;83(1); 10.1128/aem.02305-16. 10.1128/AEM.02305-16 PMC5165125 27815276

[30] Castillo D, Kauffman K, Hussain F, Kalatzis P, Rorbo N, Polz MF, et al. Widespread distribution of prophage-encoded virulence factors in marine Vibrio communities. Scientific Reports. 2018;8; 10.1038/s41598-018-28326-9. 10.1038/s41598-018-28326-9 PMC6028584 29967440

[31] Helgason E, Okstad OA, Caugant DA, Johansen HA, Fouet A, Mock M (2000). Bacillus anthracis, Bacillus cereus, and Bacillus thuringiensis–one species on the basis of genetic evidence. Appl Environ Microbiol.

[32] Tzipilevich E, Habusha M, Ben-Yehuda S (2017). Acquisition of phage sensitivity by bacteria through exchange of phage receptors. Cell..

[33] Stokar-Avihail A, Tal N, Erez Z, Lopatina A, Sorek R (2019). Widespread utilization of peptide communication in phages infecting soil and pathogenic bacteria. Cell Host Microbe..

[34] Li XX, Zhang C, Wei FC, Yu F, Zhao Z. Bactericidal activity of a holin-endolysin system derived from *Vibrio alginolyticus* phage HH109. Microbial Pathogenesis. 2021;159; 10.1016/j.micpath.2021.105135. 10.1016/j.micpath.2021.105135 34390766

[35] Chang Y. Bacteriophage-derived endolysins applied as potent biocontrol agents to enhance food safety. Microorganisms. 2020;8(5); 10.3390/microorganisms8050724. 10.3390/microorganisms8050724 PMC7285104 32413991

[36] Besemer J, Borodovsky M (2005). GeneMark: web software for gene finding in prokaryotes, eukaryotes and viruses. Nucleic Acids Res.

[37] Moraru C, Varsani A, Kropinski AM. VIRIDIC-A novel tool to calculate the intergenomic similarities of prokaryote-infecting viruses. Viruses-Basel. 2020;12(11); 10.3390/v12111268. 10.3390/v12111268 PMC7694805 33172115

[38] Aziz RK, Bartels D, Best AA, DeJongh M, Disz T, Edwards RA (2008). The RAST Server: rapid annotations using subsystems technology. BMC Genomics.

[39] Darling ACE, Mau B, Blattner FR, Perna NT (2004). Mauve: multiple alignment of conserved genomic sequence with rearrangements. Genome Res.

